# Predisposing, enabling and need factors associated with skilled delivery care utilization among reproductive-aged women in Kersa district, eastern Ethiopia

**DOI:** 10.1186/s12978-019-0829-z

**Published:** 2019-11-15

**Authors:** Gezahegn Tesfaye, Catherine Chojenta, Roger Smith, Deborah Loxton

**Affiliations:** 1 School of Public Health, College of Health and Medical Sciences, Haramaya University, P.O Box: 235, Harar, Ethiopia; 2 Research Centre for Generational Health and Ageing, Faculty of Health and Medicine University of Newcastle, Newcastle upon Tyne, NSW Australia; 3 Mothers and Babies Research Centre, Faculty of Health and Medicine, University of Newcastle, Newcastle, NSW Australia

**Keywords:** Skilled delivery care, Predisposing factors, Enabling factors, Need factors, Reproductive-aged women, Kersa district, Eastern Ethiopia

## Abstract

**Background:**

Skilled delivery care utilization in Ethiopia is still very low compared with the goal set by the global community for countries with the highest maternal mortality. As a result, the country is overburdened with high maternal morbidity and mortality. We aimed to explore the predisposing, enabling, and need factors associated with skilled delivery care utilization among reproductive-aged women in Kersa district, eastern Ethiopia.

**Methods:**

A community-based cross-sectional study was conducted with a total of 1294 women. The participants were selected using systematic sampling techniques. An interviewer-administered structured questionnaire aided by an electronic survey tool was used to collect data. Univariate analyses were conducted to describe the study sample. Bivariate and multivariate logistic regression analyses were carried out to elicit the association of predisposing, enabling, and need factors associated with skilled delivery care utilization. Separate multivariate models were fitted for primiparous and multiparous women categories. Odds ratios with 95% confidence intervals were used to assess statistical significance.

**Results:**

More than a quarter (30.8%) of the women surveyed used skilled delivery care for their most recent birth. Significant predisposing factors were as follows: presence of educated family member; receiving education on maternal health; previous use of skilled delivery care; and best friend’s use of maternal care. Place of residence was the enabling factor that predicted skilled delivery care use. Antenatal care attendance and pregnancy intention were significant need factors associated with skilled delivery care utilization.

**Conclusion:**

The findings of the study highlight the need for a concerted effort to establish community-based peer education programs; improve access to family planning services (to reduce unintended pregnancies); increase antenatal care uptake; and facilitate access to skilled delivery care in rural areas.

## Plain English summary

Giving birth with the assistance of skilled health care professionals is referred to as skilled delivery care. Skilled delivery care is important to prevent maternal deaths that result from birth-related complications. Increasing the use of skilled delivery care is advocated as an important strategy to reduce maternal mortality. However, many women in developing countries such as Ethiopia do not wish to give birth at health facilities attended with skilled health care professionals. In Ethiopia, nearly three-quarters of women give birth at home, and the rate of skilled delivery care utilization is very low. The purpose of this study was to identify the key factors that are associated with skilled delivery care utilization in a largely rural district in Eastern Ethiopia. We conducted a community based cross-sectional study which involved a face to face interview with eligible women. A total of 1294 eligible women completed the interview. Data were summarized descriptively, and the key factors associated with the use of skilled delivery care were identified using regression analysis. The results indicated that more than a quarter of the women attended skilled delivery care for their last birth. Having an educated family member, receiving maternal health education, previous use of skilled delivery care, having a best friend who used maternal care, urban residence, intended pregnancy, and antenatal care use during their last pregnancy were facilitating factors for skilled delivery care utilization. These findings highlight the need to institute measures that include these factors to improve the use of skilled delivery care.

## Introduction

Globally, an estimated 303,000 women died due to pregnancy and childbirth complications in 2015, with developing countries accounting for nearly 99% of the global burden [1]. Although most maternal deaths that result from pregnancy complications are avoidable; all pregnancies that continued to birth could face obstetric complications that might not be identified during antenatal care (ANC) check-ups. Hence, the presence of a skilled health worker at birth for every delivery remains a crucial factor for the improvement of obstetric outcomes [2] and prevention of maternal deaths that occur during delivery [3]. Up to one-third of maternal mortality could be prevented by the appropriate management of birth complications by skilled health workers during birth [4].

A review of studies in Ethiopia [5] identified very low utilization of professionally assisted delivery care at a health facility as one of the main contributing factors to the existing high burden of maternal deaths in the country. Hence, promoting skilled delivery care attendance at health facilities is an essential strategy to prevent maternal mortality [6, 7].

Skilled delivery care attendance is low in most developing countries, with some of the lowest rates of attendance in sub-Saharan African countries [8], where the majority of maternal deaths occur [9]. In Ethiopia, according to a recent Demographic and Health Survey report, the proportion of women who utilized skilled birth assistance during delivery was 28% [10]. However, the global community has set a goal of reaching 60% utilization of skilled delivery care attendance during birth for countries with the highest maternal mortality, and 90% at a global level [11]. Based on the trend of data in the two decades before 2010, the estimated rate of annual improvement in the utilization of skilled delivery care in Ethiopia between the years 2011 to 2015 is 0.4%, which is also far lower than the average rate of improvement in other sub-Saharan African countries [12]. Many other studies [13–18] on skilled delivery care utilization in Ethiopia found less than 40% of pregnant women accessed these services.

A multitude of factors including place of residence, education, wealth status, pregnancy intention, parity, birth order, previous use of care, physical distance, and cultural barriers are associated with skilled delivery care utilization [19–26]. However, there is limited evidence as to the contextual personal, social, and community factors that motivate a majority of pregnant women in Ethiopia to avoid delivering their babies with the assistance of skilled health workers. The existing evidence is restricted to identifying the comprehensive factors influencing skilled delivery care utilization and does not reflect the underlying regional variations in socio-economic, geographic, and cultural influences. Consequently, there is a need to understand the regional level contextual factors operating as barriers in the utilization of skilled delivery care, to better inform context-specific interventions. In this study, we attempt to understand the factors associated with skilled delivery care attendance by using the Andersen and Newman behavioural model of health care utilization [27, 28]. This model typically classifies the factors that influence utilization of health care into three categories. The *predisposing factors* are the demographic and social conditions that influence the person’s decision to use the service. The *enabling factors* are economic circumstances that facilitate service utilization. The *need factors* reflect the perceived health service needs and are related to the actual illness condition. The aim of this study was to assess the influence of these three factors on skilled delivery care utilization among reproductive-aged women in Kersa district, eastern Ethiopia.

## Methods

### Study area and period

The study was conducted in Kersa district, East Hararghe zone, Oromia region, eastern Ethiopia. Kersa town, the capital of the district, is located 486 kms from Addis Ababa, the capital city of Ethiopia. The total population of the district was 205,628, as of the 2014 population projection for Ethiopia. In the district, there were 38 *kebeles* (the lowest administrative unit in Ethiopia consisting of 5000 people or 1000 households) or so-called subdistricts. From the 38 *kebeles*, three were urban, and 35 were rural [29, 30], and 24 *kebeles* were under the Health and Demographic Surveillance System (HDSS). There were seven health centres, 34 health posts and eight private pharmacies in the district. The health centres routinely provide the recommended packages of ANC, skilled delivery care and postnatal care. Based on a recent report from the district health office, the health coverage of the district was 80% [31]. The study was conducted from June to August 2017.

### Study design

A community-based cross-sectional study was implemented.

### Population

The population for the study were all reproductive-aged women living in the Kersa district. Only women who had at least one birth within the previous 3 years, had lived in the district for more than 6 months, and had delivered their most recent baby after 28 weeks of gestation were included. Women who were critically ill, and physically or mentally disabled were excluded from the study.

### Sample size and sampling procedure

The total sample size for the study (*n* = 1320) was primarily calculated for a study on maternal health service utilization and associated factors in Kersa district, eastern Ethiopia using different parameters. Twenty-five percent of the total 38 *kebeles* in the district (i.e. 10 *kebeles*) were included in the study to ensure optimum sample representation. Of the included *kebeles*, two were urban, and eight were rural. The study district was first stratified into urban and rural *kebeles*, and these were further classified into HDSS and non-HDSS *kebeles*. A proportional number of study *kebeles* were then selected from each stratum using simple random sampling technique. To select the individual study participants, first, the number of households having at least one eligible woman was determined in each *kebele* using the Health Extension Workers’ logbook. Since the number of households with eligible women varied among the included *kebeles,* the total sample size of the study was proportionally allocated to each selected *kebele*. The total calculated sample size was then proportionally allocated to each *kebele* based on the determined number of households with eligible women for each *kebele*. The study participants were drawn from the list of households having eligible women using systematic random sampling techniques. The participants were recruited at the time of the survey based on a pre-identified list of randomly selected households. In the event of two or more eligible women being in the same household, only one woman was randomly selected and interviewed to avoid intra-household correlations.

### Measurement variables

#### Outcome variable

**Skilled delivery care utilization:** women who have received delivery care from a skilled health worker (doctors, midwives/nurses, or health officers) at the time of labour and parturition of their most recent baby irrespective of the setting in which the birth occurs.

#### Covariates

(i) *Predisposing factors:* Woman’s age; educational status; partner’s educational level; presence of another educated family member; age at marriage; birth order; age at first pregnancy; education about maternal health; previous use of skilled delivery care; living in model family; best friend’s use of maternal care; plus mass media and telephone availability at the household level.
(ii) *Enabling factors*: Residence; wealth index; decision making on household expenses; proximity to the nearest health facility; social support; and, gender of the household head.
(iii) *Need factors*: Health Extension Workers household visits; experience of still-birth; ever had an abortion; pregnancy intention; ANC attendance for the index pregnancy; history of infant death; and, experience of delivery complications.

### Source of data and data collection methods

A supervisor and eight enumerators were involved in the data collection process. The data were collected using a structured questionnaire administered by face to face interviews at the participant’s home. The survey questionnaire was adapted from relevant literature [32–36] that addresses maternal health service utilization. Variables were measured using closed ended questions and participants were queried about basic socio-demographic variables; reproductive histories; primary health care services; health promotion and women’s autonomy; pregnancy status and maternal health service utilization; social network; and, social support. Using the translation-back-translation method, first, the original English version of the questionnaire was translated to the local language, Oromiffa. The Oromiffa version was then translated back into English by a translator who was not involved in the first phase of the translation process. The interviews were conducted using the local language. An off-line mode of digital data collection software (Survey Gizmo) installed on iPads was used to collect the responses. Kersa HDSS resident enumerators fluent in the local language conducted the individual interviews. A field supervisor and the lead author closely monitored the data collection at the field level.

### Data quality control

Pre-testing of the tool was carried out on 5% of the sample of women in the adjacent district. Necessary refinements were made on the tool based on the pre-test findings. We recruited and employed experienced local data collectors and a supervisor. Intensive training was provided to the data collectors and the supervisor on the objectives of the study, methodology, and sampling procedures and how to use the iPads for collecting responses. The use of iPads during data collection prevented the potential for incomplete responses and missing values. At the field level, 10% of the interviewed women were re-interviewed by the supervisor to check the validity of the responses. During the quality check, if the supervisor found any invalid response on the Survey Gizmo, the interviewer revisited the house and interviewed the woman again. The enumerators uploaded the responses daily to the online version of Survey Gizmo. The responses were then double-checked daily by the lead author for any inconsistencies.

### Data management and analysis

Data were analyzed using SPSS version 23 software package. We conducted the transformation of some of the variables to allow for undertaking a meaningful analysis. For the transformation, we used either recoding (through collapsing categories of some nominal variables and categorizing continuous variables) or creating new variables from the existing ones using statistical computation techniques. Univariate analyses were conducted to descriptively summarize the characteristics of the sample population. The existence of multicollinearity between covariates was determined using a Variance Inflation Factor value less than five. The multivariate model fitness was verified using Hosmer-Lemeshow test. Bivariate logistic regression analysis was carried out to compare utilization of skilled delivery care among different groups using *p*-value. Variables that showed statistical association at a p-value less than 0.05 from each set of variables in the bivariate analysis were entered into the final full multivariate logistic regression model. Two multivariate logistic regression models were fitted (*Model 1* for multiparous women and *Model 2* for primiparas women) to identify factors associated with skilled delivery care utilization. The measure of association using Adjusted Odds Ratios (AOR) with Confidence Intervals (CI) was used to assess the direction as well as the strength of the association between the explanatory and the outcome variables.

### Ethical considerations

The study was conducted after securing ethical approval from the Human Research Ethics Committee of the University of Newcastle, Australia and the Institutional Health Research Ethics Review Committee of College of Health and Medical Sciences, Haramaya University, Ethiopia. Informed verbal consent was obtained from each respondent before commencing the interviews. The confidentiality of the respondents was ensured by avoiding personal identification details. During house-to-house interviews, the participants’ privacy was maintained by carrying out the interviews in a separate place in their residence where auditory and visual privacy was assured.

## Result

### Socio-demographic characteristics

In this study, of the 1320 women who were approached, a total of 1294 women gave complete responses for the interviews. Out of these, 50.4% were aged 25–34 years with a mean age of 27.4 (±6) years. The dominant ethnicity and religion were Oromo (98.5%) and Muslim (96.8%) respectively. Married women were nearly 98.7% of the sample, and about one quarter had at least an elementary school education (Table 1).

**Table 1:** Distribution of socio-demographic characteristics by place of residence among women in Kersa district, eastern Ethiopia, 2017

Variables	Rural	Urban	Total
	*N* (%)	*N* (%)	*N* (%)
Age (*n* = 1294)			
15–24	353 (31.5)	58 (33.1)	411 (31.8)
25–34	558 (49.9)	94 (53.7)	652 (50.4)
35–49	208 (18.6)	23 (13.1)	231 (17.9)
Marital status (*n* = 1294)			
Married	1105 (98.7)	172 (98.3)	1277 (98.7)
Divorced	4 (0.4)	1 (0.6)	5 (0.4)
Never married	1 (0.1)	2 (1.1)	3 (0.2)
Separated	2 (0.2)	0 (0.0)	2 (0.2)
Widowed	7 (0.6)	0 (0.0)	7 (0.5)
Wealth index (*n* = 1294)			
Highest	208 (18.6)	50 (28.6)	258 (19.9)
Fourth	204 (18.2)	57 (32.6)	261 (20.2)
Middle	228 (20.4)	32 (18.3)	260 (20.1)
Second	243 (21.7)	15 (8.6)	258 (19.9)
Lowest	236 (21.1)	21 (12.0)	257 (19.9)
Ethnicity (*n* = 1294)			
Oromo	1118 (99.9)	156 (89.1)	1274 (98.5)
Amhara and Arab	1 (0.1)	19 (10.9)	20 (1.5)
Religion (*n* = 1294)			
Muslim	1115 (99.6)	138 (78.9)	1253 (96.8)
Protestant and Orthodox	4 (0.4)	37 (21.1)	41 (3.2)
Occupational status (*n* = 1294)			
Housewife	1099 (98.2)	141 (80.6)	1240 (95.8)
Government employee	1 (0.1)	23 (13.1)	24 (1.9)
Merchant	8 (0.7)	11 (6.3)	19 (1.5)
Farmer	11 (1.0)	0 (0.0)	11 (0.9)
Educational status (*n* = 1294)			
Never attended	889 (79.4)	52 (29.7)	941 (72.7)
Elementary	205 (18.3)	62 (35.4)	267 (20.6)
Secondary	23 (2.1)	38 (21.7)	61 (4.7)
Tertiary	2 (0.2)	23 (13.1)	25 (1.9)
Partner’s education (*n* = 1279)			
Never attended	620 (55.4)	25 (14.3)	645 (49.8)
Elementary	394 (35.2)	44 (25.1)	438 (33.8)
Secondary	88 (7.9)	45 (25.7)	133 (10.3)
Tertiary	5 (0.4)	58 (33.1)	63 (4.9)
Partner’s occupation (*n* = 1279)			
Farmer	1090 (97.4)	66 (37.7)	1156 (89.3)
Government employee	6 (0.5)	59 (33.7)	65 (5.0)
Merchant	7 (0.6)	22 (12.6)	29 (2.2)
Daily labourer	4 (0.4)	25 (14.3)	29 (2.2)
Educated family member (*n* = 1294)			
Never attended	672 (60.1)	48 (27.4)	720 (55.6)
Elementary	366 (32.7)	70 (40.0)	436 (33.7)
Secondary	75 (6.7)	35 (20.0)	110 (8.5)
Tertiary	6 (0.5)	22 (12.6)	28 (2.2)

### Reproductive characteristics of the women

More than three-fourths (83.3%) of the women had been pregnant more than once, and 1059 (81.8%) of the women had given birth more than once. Small numbers of respondents, 95 (7.4%), 94 (7.3%), and 264 (20.4%) had ever experienced still-birth, abortion and infant death respectively. More than a quarter of the women (29.1%) admitted that their most recent pregnancy was unintended. About 1114 (86.1%) of the women were first married when they were less than 18 years of age, and 1155 (89.3%) of the women became pregnant for the first time at the age of 20 years or less. For 671 (51.9%) of the women, their most recent child was their third or less pregnancy, and for 1275 (98.5%) of the women, their most recent child was a full-term delivery. More than half (693 or 53.6%) of the women had attended at least one ANC for their most recent pregnancy (Table 2).

**Table 2:** Reproductive characteristics of the women, Kersa district, eastern Ethiopia

Variables (*n* = 1294)	Frequency	Percentage
Age of marriage		
< 18 years	1114	86.1
≥18 years	180	13.9
Age at first pregnancy		
≤ 20 years	1155	89.3
> 20 years	139	10.7
Birth order		
≤3rd	671	51.9
>3rd	623	48.1
Birth outcome of the last child		
Live full term	1275	98.5
Live preterm	12	0.9
Stillbirth	7	0.5
Gravidity		
Primigravida	216	16.7
Multigravida	1078	83.3
Parity		
Primipara	235	18.2
Multipara	1059	81.8
History of stillbirth		
No stillbirth	1199	92.6
Had stillbirth	95	7.4
History of abortion		
No history of abortion	1200	92.7
Had a history of abortion	94	7.3
History of infant death		
No history of infant death	1030	79.6
Had a history of infant death	264	20.4
Pregnancy intention for last birth		
Intended	917	70.9
Unintended	377	29.1
ANC use (at least one visit)		
Yes	693	53.6
No	601	46.4

### Skilled delivery care utilization

The utilization of skilled delivery care for the pregnancy or pregnancies prior to the most recent was 20.1%. The proportion of women who attended skilled delivery care for the most recent birth was 30.8% regardless of the place of birth, whilst 384 (29.7%) of the births were assisted by a skilled health provider at a health facility. Slightly under half (194 or 48.9%) of the women opted for health facility delivery because it was close to where they lived; whilst almost a quarter (91 or 22.9%) cited high-quality services as a motivating factor. Among the women who delivered at the health facility, the final decision to deliver there was made by the respondent themselves (49.1%), followed by their husbands (21.7%). About 235 (59.2%) of the women did not make any arrangement or birth plan prior to delivering the most recent child. A high proportion (305 or 76.8%) of the women, were accompanied to a health facility for delivery by their partner, followed by traditional birth attendants (53 or 13.4%) and Women’s Development Army leaders (50 or 12.6%) (Table 3).

**Table 3:** Skilled delivery care utilization among respondents, Kersa district, eastern Ethiopia

Variable	Frequency	Percentage
Skilled delivery care for previous pregnancies (*n* = 1059)		
Yes	213	20.1
No	846	79.9
Skilled delivery care for the recent pregnancy (*n* = 1294)		
Yes	398	30.8
No	896	69.2
Place of delivery for the recent pregnancy (*n* = 1294)		
Government hospital	71	5.5
Government health centre	306	23.6
Government health post	17	1.3
Private hospital/clinic	3	0.2
Home	890	68.8
Other (e.g. on the road)	7	0.5
Reason to prefer that facility for delivery (*n* = 397**)		
Close to where I live	194	48.9
Helpfulness of health workers	79	19.9
High-quality services	91	22.9
Little or no expenses	21	5.3
Other reasons	12	3.02
Person attending the care at the health facility (*n* = 397)		
Doctor	47	11.8
Health officer	11	2.8
Nurse/midwife	326	82.2
Health Extension Worker	13	3.3
Who made final decision to deliver at the health facility (*n* = 397)		
Yourself	195	49.1
Your husband	86	21.7
Jointly with your husband	45	11.5
Health Extension Worker	41	10.3
Your parents	14	3.5
Mother-in-law	6	1.5
Women’s Development Army leader	5	1.3
Other	5	1.3
Who accompanied to the health facility (*n* = 397)		
Partner	305	76.8
Women’s Development Army leader	50	12.6
Health Extension Workers	20	5.0
Traditional birth attendants	53	13.4
No one	32	8.1
Birth preparedness (*n* = 397)		
Yes	156	39.3
No	235	59.2
Don’t remember	6	1.5

**The total number of respondents who delivered at a health facility. The number of women who received skilled care from either a (doctor, nurse/midwife, or health officer) is 398 regardless of the place of delivery. The number of women who were assisted with skilled personnel within a facility is 384. [Birth preparedness: An advance preparation and planning for birth by the woman and her family]

### Referral, prior plan for facility birth and delivery complications

Among the women who presented for delivery at the health facility, 46 (11.6%) were referred to a higher-level health facility for delivery. More than three-quarters (317 or 79.8%) of the women had a prior plan for facility delivery for their most recent birth. Only 94 (7.3%) of the women experienced obstetric complications during the delivery of their most recent baby, with prolonged labour (36.2%) and severe bleeding (29.8%) being the most common types of complications. Out of those women who delivered their most recent baby at a health facility, 90 (22.7%) did not recommend facility delivery to their friends. A significant proportion of women (364 or 28.1%) still wished to deliver their next baby at home (Table 4).

**Table 4:** Referral, prior plan for facility birth, and complications during delivery among women in Kersa district, eastern Ethiopia, 2017

Variable	Frequency	Percentage
Referral to a higher facility (*n* = 397)		
Yes	46	11.6
No	351	88.4
Prior plan for health facility delivery (*n* = 397)		
Yes	317	79.8
No	73	18.4
Don’t know	7	1.8
Presence of complication during delivery (*n* = 1294)		
Yes	94	7.3
No	1197	92.5
Don’t know	3	0.2
Type of complications during delivery* (*n* = 94)		
Severe bleeding	28	29.8
Severe headache	25	26.6
Convulsions	8	8.5
High fever	12	12.8
Loss of consciousness	13	13.8
Labour lasting > 12 h	34	36.2
Placenta not delivered 30 min after the baby	14	14.9
Other (abdominal pain and severe vomiting)	2	2.1
Place where complication developed (*n* = 94)		
Home	63	67.1
Health facility	29	30.9
On the way to a health facility	2	2.1
Sought assistance for complication during delivery (*n* = 94)		
No	35	37.2
Yes	59	62.8
Future choice of delivery (*n* = 1294)		
Health facility	764	59.0
Home	364	28.1
No plan for pregnancy	166	12.8
Recommend facility delivery to friends (*n* = 397)		
Yes	307	77.3
No	90	22.7

*Does not sum up to total due to the possibility of multiple responses

### Factors associated with skilled delivery care utilization

#### Predisposing factors

Multiparous women who had educated family members had an increased odds of skilled delivery care attendance (AOR, 1.89; 95% CI: 1.26, 2.85) than women who had no educated family members. Primipara women who had received education on maternal health had higher odds of using skilled delivery care (AOR, 2.22; 95% CI: 1.09, 4.53) than those who had not received maternal health education. Multiparous women with no previous experience of receiving skilled delivery care had lower odds of skilled delivery care utilization for subsequent births (AOR, 0.08; 95% CI: 0.05, 0.13) than their counterparts. Multiparous women whose best friends did not use maternal care had decreased odds of skilled delivery care use (AOR, 0.43; 95% CI: 0.29, 0.64) than multiparous women whose best friends used maternal care.

#### Enabling factors

Place of residence was significantly associated with skilled delivery care attendance in both multivariate logistic regression models. Urban residence had significantly increased the odds of skilled delivery care utilization ((AOR, 4.83; 95% CI: 2.58, 9.05) in *Model 1* and (AOR, 8.23; 95% CI: 2.26, 29.97) in *Model 2*).

#### Need factors

Multiparous women with unintended pregnancies had a decreased odds of skilled delivery care utilization (AOR, 0.54; 95% CI: 0.35, 0.84) than multiparous women with intended pregnancies. In both multivariate models, ANC attendance during the most recent pregnancy was positively associated with skilled delivery care utilization during the most recent birth ((AOR, 2.23; 95% CI: 1.48, 3.37) in *Model 1* and (AOR, 3.13; 95% CI: 1.51, 6.46) in *Model 2*) (Table 5).

**Table 5:** Factors associated with the utilization of skilled delivery care among women in Kersa district, eastern Ethiopia, 2017

Variables	Categories	Skilled delivery care use *(n = 1059) Model 1* *(Multiparous women)*		Skilled delivery care use *(n = 235) Model 2* *(Primiparous women)*	
		COR (95% CI)	AOR (95% CI)	COR (95% CI)	AOR (95% CI)
Predisposing factors					
Educational status	Never attended	1	1	1	1
	Attended	3.55 (2.62, 4.80)	1.37 (0.84, 2.22)	4.89 (2.81, 8.50)	1.09 (0.50, 2.38)
Partner’s educational level	Never attended	1	1	1	1
	Attended	2.06 (1.57, 2.72)	0.86 (0.58, 1.29)	4.50 (2.52, 8.03)	1.97 (0.91, 4.27)
Presence of another educated family member	Yes	2.05 (1.55, 2.70)	**1.89 (1.26, 2.85)**	5.77 (2.95, 11.29)	1.36 (0.51, 3.61)
	No	1	**1**	**1**	1
Educated about maternal health	Yes	1.50 (1.14, 1.97)	1.17 (0.80, 1.71)	2.66 (1.57, 4.51)	**2.22 (1.09, 4.53)**
	No	1	1	1	1
Mass media availability at home	Yes	1.98 (1.50, 2.62)	0.99 (0.64, 1.55)	2.30 (1.36, 3.89)	0.88 (0.41, 1.90)
	No	1	1	1	1
Telephone ownership at household level	Yes	2.37 (1.76, 3.19)	1.07 (0.66, 1.74)	4.03 (2.30, 7.08)	1.58 (0.69, 3.62)
	No	1	1	1	1
Birth order	≤3rd	1	1		
	>3rd	0.59 (0.45, 0.78)	0.76 (0.49, 1.18)		
Previous use of skilled delivery care	Yes	1	1		
	No	0.06 (0.04, 0.08)	**0.08 (0.05, 0.13)**		
Living in a model family	Yes	1	1		
	No	0.43 (0.22, 0.87)	1.76 (0.62, 5.02)		
Best friend’s use of maternal care	Yes	1	1	1	1
	No	0.22 (0.16, 0.29)	**0.43 (0.29, 0.64)**	0.25 (0.14, 0.43)	0.77 (0.38, 1.56)
Enabling factors					
Residence	Rural	1	1	1	1
	Urban	14.42 (9.09, 22.88)	4.83 (2.58, 9.05)**	23.71 (8.18, 68.73)	8.23 (2.26, 29.97)**
Wealth index	Highest	1.88 (1.21, 2.90)	1.80 (0.98, 3.32)		
	Fourth	1.72 (1.11, 2.67)	1.45 (0.80, 2.64)		
	Middle	1.37 (0.88, 2.24)	1.63 (0.90, 2.94)		
	Second	0.83 (0.52, 1.32)	0.81 (0.44, 1.49)		
	Lowest	1	1		
Decision making on household expenses	Respondent	1	1	1	1
	Jointly	1.10 (0.75, 1.61)	0.69 (0.42, 1.12)	2.49 (1.07, 5.78)	1.83 (0.67, 4.97)
	Partner	1.95 (1.20, 3.18)	1.65 (0.84, 3.23)	1.83 (0.70, 4.79)	2.90 (0.87, 9.71)
Social support from friends	Yes	1	1	1	1
	No	0.34 (0.18, 0.62)	0.58 (0.27, 1.25)	0.30 (0.14, 0.64)	0.71 (0.29, 1.76)
Need factors					
History of infant death	Have no history	1	1		
	Have history	0.68 (0.49, 0.95)	1.09 (0.70, 1.72)		
Pregnancy intention	Intended	1	1		
	Unintended	0.50 (0.37, 0.69)	**0.54 (0.35, 0.84)**		
ANC attendance for the most recent pregnancy	Yes	5.10 (3.70, 7.02)	2.23 (1.48, 3.37)**	6.96 (3.86, 12.53)	3.13 (1.51, 6.46)**
	No	1	1	1	**1**

*Key: COR* Crude Odds ratio, *AOR* Adjusted Odds ratio (adjusting for the potential predisposing, enabling and need factors in full model), *CI* Confidence Interval (95%), Bold*statistically significant variables, Bold**statistically significant variables in both models (*Model 1 and 2*)

## Discussion

This study has attempted to explore the factors that influence the uptake of skilled delivery care among reproductive-aged women in a predominantly rural district in eastern Ethiopia; and definitively quantify use. Having an educated family member; receiving education on maternal health; previous use of skilled delivery care; and, best friend’s use of maternal care were significantly associated predisposing factors. Place of residence was the only significantly associated enabling factor. The significant need factors were pregnancy intention and ANC attendance for the most recent pregnancy.

The results of the current study showed that the utilization of skilled delivery care among reproductive-aged women was 30.8%. This figure reflects findings of an Ethiopian national Demographic and Health Survey report [10], where 28% of the women delivered their most recent baby with the assistance of a skilled provider; as well as a study conducted in Southern Ethiopia [18] in which 31% of women utilized skilled delivery care during their delivery. However, our result is higher than several other studies in Ethiopia [14, 15, 37, 38]. The relatively higher level of skilled delivery care utilization in our study may reflect current efforts to reduce home delivery using financial penalties for traditional birth attendants and local pregnant women who opt for home births. Moreover, the evolution of the Women’s Development Army network at the village level might have contributed to better uptake of skilled delivery care through health discussions within the network during regular meetings. The varying level of service uptake underscores the requirement for local-level research to highlight the setting-specific factors that either impede or facilitate service utilization, thereby informing the development of locally suitable programs.

The findings of the present study demonstrate that the predisposing factors associated with skilled delivery care utilization were: having an educated family member: receiving education on maternal health: previous use of skilled delivery care: and, best friend’s use of maternal care. Multiparous women who have an educated family member in their household have increased odds of using skilled delivery care. The influence that the educated family member had on the woman’s acceptance of utilizing material care is paramount. It has been shown [39] that living in close proximity to people with a higher level of education can strongly influence the health service seeking behaviour of women. People with more education tend to have healthier behaviours, and good knowledge and understanding about the importance of health care utilization [40]. In relation to this, primiparous women who had received education on maternal health have increased odds of skilled delivery care utilization than primiparous women who had not received maternal health education. Educating women about maternal health increases their understanding of the importance of attending maternal health services; and the possible catastrophic consequences of pregnancy or birth complications; contributing to their acceptance and uptake of skilled delivery care. This positive impact of maternal health knowledge on the uptake of skilled delivery care has been evidenced in a previous study in Nepal [41].

Previous experience of using skilled delivery care was significantly associated with skilled delivery care utilization. The finding may indicate that women perceived the delivery care services they had received during the previous birth to be quality and appropriate. The association of previous care experience with future use of skilled delivery care might also reflect the confounding effect of accessibility and availability of services. The women who previously used skilled delivery care that subsequently attend the same service might already have better accessibility to the service, which continues to be available for the subsequent births, unlike women who do not use the service. However, in a study conducted in northwest Ethiopia [42] contrasting results were found, in that previous attendance of delivery care had a limited role in increasing the uptake of skilled delivery care for the subsequent birth. This difference may highlight the variation in terms of quality of care at different levels of primary health care facilities in Ethiopia. Moreover, in Ethiopia, concerted efforts should be made to improve the quality of obstetric care at health facilities to increase the utilization of skilled delivery care.

Our findings showed that the best friend’s non-use of maternal care is significantly associated with poor uptake of skilled delivery care. The result was comparable with a study conducted in Mali [39], where women’s health-seeking and utilization behaviour towards maternal care use was significantly influenced by the care-seeking practices of the surrounding people in their areas of residence. Friends are often connected through providing moral support to one another which influences the decision either to refuse or accept a temptation [43]. Women are more likely to adopt unhealthy/healthy behaviours from their best friends than strangers [44], especially in regard to gendered issues they experience in common, such as pregnancy or birthing. During their interactions with friends, fear and stigma are decreased, allowing women to freely discuss their delivery choices and therefore a adopt bad or good care-seeking behaviour. This implies that there is a possible benefit in organizing maternal health programs that target networks at the community level, as opposed to individuals. The Women’s Development Army network, already established in most districts of Ethiopia, is an ideal platform and resource to facilitate such programs. Peer education programs customised specifically for women’s social networks at the village level have great potential to accelerate improved attendance of skilled delivery care.

We also revealed in this study that place of residence is a significantly associated enabling factor for skilled delivery care utilization. Urban women have increased odds of utilizing skilled delivery care compared to rural women. The association of urban residence with better uptake of maternal care is consistent with the findings from other studies in Ethiopia [18, 20, 42, 45], and elsewhere in sub-Saharan Africa [46, 47] where the odds of skilled delivery care utilization is very low among rural-dwelling women compared to urban women. Better awareness of, and access to, maternal care may account for much of the difference between urban and rural women’s uptake of skilled delivery care. Additionally, as urban women generally tend to have a higher level of economic status, they can better afford the financial burden associated with utilization of the service. Local efforts to improve maternal health should, therefore, focus on improving the accessibility and availability of delivery care services during birth, and the equitable distribution of health resources in urban and rural settings.

Moreover, ANC attendance for the index pregnancy was a statistically significant need factor that was associated with skilled delivery care utilization. Women who attended ANC for the index pregnancy have higher odds of attending skilled delivery care compared to women who did not attend ANC. The finding is in agreement with a swathe of other studies in Ethiopia [14, 20, 42, 48, 49] where the use of ANC for the woman’s most recent pregnancy was strongly associated with an increased likelihood of attending skilled delivery care for the most recent birth. The women had physical interactions with the health workers and the facility during ANC that might, in turn, have helped them become more confident and familiar with the health facility allowing them to seek skilled delivery care [39]. Moreover, during the ANC check-up, the women had the opportunity to receive advice and information related to the benefits of skilled delivery care as well as the potential occurrence of obstetric problems during delivery which could increase their perceived susceptibility and motivate them to attend the care [37, 50, 51].

Women with unintended pregnancies have lower odds of skilled delivery care utilization compared to women with intended pregnancies. These findings are consistent with those of a previous Ethiopian study [19], which showed that skilled delivery utilization is higher among women who have intended rather than unintended pregnancies. The actual linking mechanism between pregnancy intentions and the use of skilled delivery and other maternal care is unclear; however, it could be related to women’s ability to having control over both their reproductive life and household resources [52].. In addition, some women with unintended pregnancies may consider abortion as an alternative to maternal health service use. Unintended pregnancies are highly indicative of an unmet need for family planning; thus, access to appropriate family planning services should be improved.

### Strengths and limitations

Limiting the reference time for retrospective questions to 3 years could minimize the introduction of recall bias. The use of relatively higher sample size has given adequate power to the study. We used a digital survey tool with iPads to collect the data, which improved the quality of the data by ensuring its completeness and consistency. Incorporation of a greater number of predictor variables into the final multivariate model ensures better controlling for confounding factors. As the interviewers were resident HDSS data collectors who regularly interact with the community, social desirability bias might not be ruled out. Due to the social desirability bias, women might have tended to provide favourable responses regarding the current or past of use of delivery care and be hesitant to share their negative care experiences. Due to the cross-sectional nature of the survey design, establishing a causality inference is also difficult. Regardless of the limitations of the study, we believe that the current survey provides a quite strong indication of the factors influencing skilled delivery care utilization in a rural setting in Ethiopia.

## Conclusion

More than a quarter of the surveyed women attended skilled delivery care for their most recent pregnancy. Place of residence; the presence of another educated family member; receiving maternal health education; previous use of skilled delivery care; best friend’s use of maternal care; pregnancy intention; and ANC attendance for the index pregnancy; were statistically significant predisposing, enabling and need factors associated with skilled delivery care utilization. The study pointed out the key areas of possible interventions for maternal health program planners. Designing a maternal health program that entails equitable distribution of health infrastructure between rural and urban communities is recommended. Moreover, safe motherhood intervention efforts should aim to improve the quality of delivery care at health facilities which in turn retains women for subsequent attendance. Improving access to family planning services; establishing peer to peer maternal health education programs; and increasing ANC utilization - an entry point for the attendance of the subsequent maternal care including skilled delivery care – are all strategies to be considered to increase maternal service uptake.

## Data Availability

The data used for this study can be made available upon reasonable request to the authors.

## Abbreviations

ANC: Antenatal Care
AOR: Adjusted Odds Ratio
CI: Confidence Interval
COR: Crude Odds Ratio
HDSS: Health and Demographic Surveillance System
SPSS: Statistical Program for Social Science
